# Supplementary material for: Using Stereochemistry to Control Mechanical Properties in Thiol–Yne Click‐Hydrogels
Source: Angew Chem Int Ed Engl. 2021 Oct 28;60(49):25856–64. doi: 10.1002/anie.202107161 (PMC9298389; doi:10.1002/anie.202107161)
Supplement: Supplementary file 1 — Supporting Information [file ANIE-60-25856-s001.pdf]

Angewandte Chemie
Eine Zeitschrift der Gesellschaft Deutscher Chemiker

Supporting Information

Using Stereochemistry to Control Mechanical Properties in Thiol–Yne Click-Hydrogels

*Laura J. Macdougall[+], Maria M. Pérez-Madrigal[+], Joshua E. Shaw, Joshua C. Worch, Christopher Sammon, Stephen M. Richardson, and Andrew P. Dove**

anie_202107161_sm_miscellaneous_information.pdf

EXPERIMENTAL SECTION

1.1 General Methods

All chemicals were purchased from Sigma-Aldrich or Fisher Scientific, unless otherwise noted, and cell culture reagents from ThermoFisher Scientific; all reagents were used without further purification unless stated otherwise.

NMR spectroscopy. All NMR spectroscopy experiments were performed at 25 °C on a Bruker DPX-300/400 NMR instrument operating at 300 or 400 MHz for ^{1}H (100.57 MHz for ^{13}C). ^{1}H NMR spectra are referenced to residual proton solvent (δ = 2.50 for DMSO–d_5) and ^{13}C NMR spectra are referenced to the solvent signal (δ = 39.52 for DMSO–d_6). The resonance multiplicities are described as s (singlet), d (doublet), t (triplet), q (quartet) or m (multiplet).

FT-IR spectroscopy. Solvent and NEt_3 were removed *in vacuo* from the organogels, and the samples were sliced into thin discs for analysis *via* FT-IR spectroscopy. Specifically, all gels were synthesised in triplicate, and sliced into 2 discs (*i.e.* 12 measurements for each unique gel). Spectra were collected using an Agilent Technologies Cary 630 FTIR spectrometer using the following conditions: 16 Scans from 600 to 4000 cm^{-1}, resolution of 4 cm^{-1}, and corrected for background absorbance. The stereochemistry was determined by normalising the *cis* C=C bending signal area at 802 cm^{-1} with the C-H stretch at 2866 cm^{-1}. When using H_2O as the solvent, this resulted in a gel product with 94% *cis* content. Therefore, the *cis* content for the remaining systems was determined by dividing the normalised peak area by the area measured for the H_2O water sample (*i.e.* 94% *cis*).

Size exclusion chromatography (SEC). PEG-hydrogel precursors were analysed using SEC performed on an Agilent 1260 Infinity II GPC/SEC System fitted with an RI and ultraviolet (UV, λ = 309 nm) detector. The polymers were eluted through an Agilent guard column (PLGel 5 µm, 50 × 7.5 mm) and two Agilent mixed-C columns (PLGel 5 µm, 300 × 7.5 mm) using DMF (buffered with 5 mM NH4BF4) as the mobile phase (flow rate = 1 mL min^{-1}, 50 °C). Number average molecular weight (M_n) and dispersity ($Đ_M = M_w/M_n$) were determined against a calibration curve based on poly(methyl methacrylate) standards (Easivial PM, Agilent).

1.2 Synthesis of 4-arm alkyne-functionalized PEG precursor (4_{2A})

All PEG precursors were prepared by an already published procedure[1] that involves simple end-group modifications highly efficient (Fischer esterification). 4-arm PEG-tetrahydroxyl (2.0 kg mol^{-1}, 10 g) was purchased from JenKem Technology, USA. To a suspension of 4-arm PEG_{2k}-OH (10 g) in benzene (75 mL) and toluene (75 mL), 2 drops of concentrated H_2SO_4 were added. The solution was heated to 80 °C with stirring to obtain a clear homogenous solution. To this solution, propiolic acid (3.6 g) was added, and the solution was heated to reflux under Dean-Stark conditions. After no more water was collected in the condenser (*ca.* 20 h), the solution was cooled to ambient temperature, and the solvents were removed *in vacuo*. The resultant oil was dissolved in CH_2Cl_2 (100 mL) and the organic phase was washed with saturated $NaHCO_3$ solution (20 mL) and brine (20 mL). The organic phase was then dried using $MgSO_4$ and stirred with charcoal (*ca.* 0.1 g) for 30 min at 40 °C. The solvent was evaporated to collect the product (4_{2A}) as light- yellow oil (6.4 g, 64%). ^{1}H NMR (300 MHz; 298 K; $(CD_3)_2CO$): δ 4.32 (t, 2H, $^3J_{H\text{-}H}$ = 6 Hz, -CH_2OCO-), 3.89 (s, 1H, -CH≡CC(O)O-), 3.71-3.74 (m, 2H, -OCH$_2$CH_2O-), 3.58 (s, 2H, -OCH_2CH_2O-), 3.42 (s, 2H, -CCH_2O). ^{1}H NMR spectroscopy indicated ca. 98% conversion of the hydroxyl group to propiolate group. SEC (DMF): M_n = 4.2 kg mol^{-1} ($Đ_M$ = 1.08).

1.3 Synthesis of 4-arm thiol-functionalized PEG precursor (4_{2S})

According to method 1.2, 4-arm PEG_{2k}–OH (10 g) was esterified using 3-mercaptopropionic acid (4.2 g) to obtain 4_{2S} (yield 7.7 g, 77%). ^{1}H NMR ($CDCl_3$, 400 MHz): δ 4.21–4.24 (t, $^3J_{HH}$ = 12, −CH_2OCO−), 3.58 (m, −OCH_2CH_2O−), 2.71–2.76 (q, $^3J_{HH}$ = 20, −OCCH_2CH_2SH), 2.63–2.66 (t, $^3J_{HH}$ = 12, −OCCH_2CH$_2$SH), 1.63–1.67 (t, $^3J_{HH}$ = 16, −SH); ^{1}H NMR spectroscopy indicated about > 99% conversion of the hydroxyl group to mercaptopropionate group. SEC (DMF): M_n= 4.7 kg mol^{-1} ($Đ_M$ = 1.06).

1.4 Synthesis of methyl-ether alkyne-functionalised PEG [PEG_{550}(C≡CH), 550 g mol^{-1}]

According to method 1.2, methyl ether PEG_{550}(OH) (molar mass 550 g mol^{-1}, 10 g) was esterified with propiolic acid (5.1 g) that resulted in a waxy solid (yield 9 g, 83%). ^{1}H NMR ($(CD_3)_2CO$, 400 MHz): δ 4.31 (t, 2H, $^3J_{HH}$ = 9 Hz, CH$_2$CH$_2$OCOCH_2), 3.88 (s, 1H, CH≡CCOO), 3.44-3.73 (m, 50H, OCH_2CH_2O), 3.28 (s, 3H, CH_3O) ppm. ^{13}C NMR ($(CD_3)_2CO$, 400 MHz): δ 153.1 (C=O), 77.7 (C≡CH), 72.43 (C≡CH), 71.0 (OCH$_2$$CH_2$O), 68.9 ($CH_2$OCO), 65.8 ($CH_2CH_2$OCO), 58.7 ($CH_3$O) ppm. FT-IR max/cm^{-1} 2869 (CH), 2112 (C≡C), 1714 (C=O), 1101 (CO). SEC ($CHCl_3$): M_n = 1.1 kg mol^{-1} ($Đ_M$ = 1.11). ^{1}H NMR spectroscopy indicated *ca.* 93% conversion of the hydroxyl group to propiolate group.

1.5 Synthesis of methyl-ether thiol-functionalised PEG [PEG_{550}(SH), 550 g mol^{-1}]

According to method 1.2, methyl ether PEG_{550}(OH) (molar mass 550 g mol^{-1}, 10 g) was esterified with 3 mercaptopropionic acid (7.6 g) (yield = 9.8 g, 85%). ^{1}H NMR ($CDCl_3$, 300 MHz): δ 3.88 (t, 2H, $^3J_{HH}$ = 8 Hz, CH_2OCO), 3.16-3.33 (m, 50H, OCH_2CH_2O), 2.99 (s, 3H, CH_3O), 2.38 (q, 2H, $^3J_{HH}$ = 12 Hz, OCCH$_2$CH_2SH), 2.33 (t, 2H, $^3J_{HH}$ = 12 Hz, OCCH_2CH$_2$SH), 1.44 (t, 1H, $^3J_{HH}$ = 8 Hz, SH) ppm. ^{13}C NMR ($(CD_3)_2CO$, 400 MHz): δ 170.4 (C=O), 71.0 (CH$_2$OCH$_3$), 69.6 (OCH$_2$CH$_2$O), 68.0 (CH$_2$OCO), 62.7 (CH$_2$CH$_2$OCO), 57.9 (CH$_3$O), 37.4 (COCH$_2$CH$_2$SH), 18.7 (COCH$_2$$CH_2$SH) ppm. FT-IR max/cm^{-1} 2869 (CH), 1734 (C=O), 1101 (CO). SEC ($CHCl_3$): M_n = 1 kg mol^{-1} ($Đ_M$ = 1.15). ^{1}H NMR spectroscopy indicated *ca.* >99% conversion of the hydroxyl group to mercaptopropionate group.

1.6 PEG $4_{2A}4_{2S}$ gels preparation and transition from organogels to hydrogels

Thiol-yne organogels were prepared with a solids content of 10 wt% by mixing solutions containing a 1:1 molar ratio of alkyne to thiol polymer precursors at ambient temperature (23 °C) using different solvent mixtures to tune the final stereochemistry of the cross-linked network. Specifically, three solvents (*i.e.* $CHCl_3$ (non-polar), acetone (moderate polarity), and H_2O (polar)) were mixed in different ratios, while triethylamine acted as the catalyst for the thiol-yne click chemistry reaction. The amount of trimethylamine added to each gelation solution, which depended on the solvent polarity, was adjusted so the gelation time for all the gels was *ca.* 1 minute regardless of the final *cis*:*trans* ratio. After mixing the precursors, the organogels cured at ambient temperature (23 °C) for 1 hour to ensure the reaction had gone to completion.

In a typical procedure, stock solutions of the required solvent mixture were prepared with NEt_3 (in the range between 0.7 and 34 µL mL^{-1}). Then, 4_{2S} (20.8 mg) and 4_{2A} (19.5 mg) were separately dissolved in 200 µL of the stock solution. Subsequently, the PEG alkyne solution was added to the PEG thiol solution, and the solution was mixed for 5 seconds in a vortex mixer; the solutions were let to gel for 1 h. After such amount of time, the gels were processed for further characterization. Only for FT-IR and Gel Fraction (GF) characterization were the gels used as

organogels, thus without solvent exchange. For all the remaining tests, the thiol-yne organogels were converted into hydrogels. To transition from an organogel to a hydrogel, $4_{2A}4_{2S}$ gels were immersed in acetone for 5 d with frequent solvent changes. Acetone was chosen since it is miscible with both $CHCl_3$ and H_2O, as well as the NEt_3 catalyst. Minimal hydrolysis occurred during this washing step. After 5 d, water was introduced gradually into the network over 2 d.

Table S1. Gelation conditions (solvent and amount of catalyst) used to prepare gels with varying *cis*:*trans* ratio.

Solvent (v/v)	NEt_3 ($\mu L\ mL^{-1}$)
H_2O	0.7
H_2O:Acetone 35:65	1.66
Acetone: H_2O 90:10	10
Acetone:$CHCl_3$ 60:40	20
$CHCl_3$	34

1.7 Determination of the *cis:trans* ratio of $4_{2A}4_{2S}$ gels by FT-IR spectroscopy

$4_{2A}4_{2S}$ gels in $CHCl_3$: the stock solution was made with $CHCl_3$ (100%) and NEt_3 at 34 $\mu L\ mL^{-1}$. FT-IR max/cm^{-1}: 2865 (CH), 1709 (C=O), 1706 (C=O), 1582 (C=C), 1094 (CO). FT-IR indicated 10% *cis* content (90% *trans*) in the gel in comparison to 94% H_2O gel (23% *cis* by ^1H NMR spectroscopy, see below for details).

$4_{2A}4_{2S}$ gels in acetone:$CHCl_3$ (60:40): the stock solution was made with acetone:$CHCl_3$ (60:40) and NEt_3 at 20 $\mu L\ mL^{-1}$. FT-IR max/cm^{-1}: 2865 (CH), 1709 (C=O), 1706 (C=O), 1582 (C=C), 1094 (CO). FT-IR indicated 23% *cis* content (77% *trans*) in the gel in comparison to 94% H_2O gel (36% *cis* by ^1H NMR spectroscopy, see below for details).

$4_{2A}4_{2S}$ gels in acetone:H_2O (90:10): the stock solution was made with acetone:H_2O (90:10) and NEt_3 at 10 $\mu L\ mL^{-1}$. FT-IR max/cm^{-1}: 2865 (CH), 1734 (C=O), 1706 (C=O), 1582 (C=C), 1094 (CO), 802 (C=C *cis*). FT-IR indicated 51% *cis* content (49% *trans*) in the gel in comparison to 94% H_2O gel (66% *cis* by ^1H NMR spectroscopy, see below for details).

$4_{2A}4_{2S}$ gels in acetone: H_2O (65:35): the stock solution was made with acetone:H_2O (65:35) and NEt_3 at 1.7 $\mu L\ mL^{-1}$. FT-IR max/cm^{-1}: 2865 (CH), 1734 (C=O), 1706 (C=O), 1573 (C=C), 1094 (CO), 802 (C=C *cis*). FT-IR indicated 82% cis content (18% *trans*) in the gel in comparison to 94% H_2O gel (91% *cis* by ^1H NMR spectroscopy, see below for details).

$4_{2A}4_{2S}$ gels in H_2O: the stock solution was made with H_2O (100%) and NEt_3 and 0.7 $\mu L\ mL^{-1}$. FT-IR max/cm^{-1}: 2865 (CH), 1734 (C=C), 1706 (C=C), 1573 (C=C), 1094 (CO), 802 (C=C *cis*). 94%

cis content assumed from ^{1}H NMR spectroscopy of small molecule reaction (93% *cis* by ^{1}H NMR spectroscopy of PEG reaction).

1.8 Determination of the *cis:trans* ratio of $4_{2A}4_{2S}$ gels by ^{1}H NMR spectroscopy

A small molecule study was employed to elucidate the relative stereochemistry of the $4_{2A}4_{2S}$ network using ^{1}H NMR spectroscopy. As a general procedure, the nucleophilic thiol-yne reaction between monofunctionalised alkyne- and thiol-PEG precursors was carried out as follow: a solution of PEG_{550}(SH), (53 mg) in the required solvent system (0.5 mL) was added dropwise to a stirring solution of PEG_{550}(C≡CH) (50 mg in 0.5 mL). The solution was stirred at ambient temperature (23 °C) for 1 h. After such amount of time, the solvent was removed *in vacuo* and the product was characterized by ^{1}H NMR spectroscopy.

Model PEG thiol-yne reaction in $CHCl_3$: ^{1}H NMR ($CDCl_3$, 300 MHz): δ 7.42 (d, 1H, $^3J_{HH}$ = 15 Hz, OCOC*H*=CHS *trans*), 6.95 (d, 1H, $^3J_{HH}$ = 12 Hz, OCOC*H*=CHS *cis*), 5.65 (d, 1H, $^3J_{HH}$ = 12 Hz, OCOCH=C*H*S *cis*), 5.55 (d, 1H, $^3J_{HH}$ = 15 Hz OCOCH=C*H*S *trans*), 4.00-4.06 (m, 2H, OCH_2CH_2OCO), 3.25-3.61 (m, 50H, OCH_2CH_2O), 3.11 (s, 3H, CH_3O) 2.42-2.51 (m, 4H, $COCH_2CH_2SH$) ppm. FT-IR max/cm^{-1} 2870 (CH), 1737 (C=O), 1709 (C=O), 1101 (CO). ^{1}H NMR spectroscopy indicated *ca.* 23% *cis* isomer selectivity.

Model PEG thiol-yne reaction in acetone:$CHCl_3$ (60:40): ^{1}H NMR ($CDCl_3$, 300 MHz): δ 7.43 (d, 1H, $^3J_{HH}$ = 15 Hz, OCOC*H*=CHS *trans*), 6.97 (d, 1H, $^3J_{HH}$ = 12 Hz, OCOC*H*=CHS *cis*), 5.68 (d, 1H, $^3J_{HH}$ = 12 Hz, OCOCH=C*H*S *cis*), 5.58 (d, 1H, $^3J_{HH}$ = 15 Hz OCOCH=C*H*S *trans*), 4.03-4.04 (m, 2H, OCH_2CH_2OCO), 3.30-3.48 (m, 50H, OCH_2CH_2O), 3.14 (s, 3H, CH_3O) 2.43-2.58 (m, 4H, $COCH_2CH_2SH$) ppm. FT-IR max/cm^{-1} 2868 (CH), 1734 (C=O), 1704 (C=O), 1099 (CO). ^{1}H NMR spectroscopy indicated *ca.* 36% *cis* isomer selectivity.

Model PEG thiol-yne reaction in acetone:H_2O (90:10): ^{1}H NMR ($CDCl_3$, 300 MHz): δ 7.42 (d, 1H, $^3J_{HH}$ = 15 Hz, OCOC*H*=CHS *trans*), 6.95 (d, 1H, $^3J_{HH}$ = 12 Hz, OCOC*H*=CHS *cis*), 5.66 (d, 1H, $^3J_{HH}$ = 12 Hz, OCOCH=C*H*S *cis*), 5.56 (d, 1H, $^3J_{HH}$ = 15 Hz OCOCH=C*H*S *trans*), 4.00-4.02 (m, 2H, OCH_2CH_2OCO), 3.27-3.64 (m, 50H, OCH_2CH_2O), 3.12 (s, 3H, CH_3O) 2.40-2.88 (m, 4H, $COCH_2CH_2SH$) ppm. FT-IR max/cm^{-1} 2866 (CH), 1735 (C=O), 1704 (C=O), 1099 (CO), 805 (C=C *cis*). ^{1}H NMR spectroscopy indicated *ca.* 66% *cis* isomer selectivity.

Model PEG thiol-yne reaction in acetone:H_2O (65:35): ^{1}H NMR ($CDCl_3$, 300 MHz): δ 7.42 (d, 1H, $^3J_{HH}$ = 15 Hz, OCOC*H*=CHS *trans*), 6.95 (d, 1H, $^3J_{HH}$ = 12 Hz, OCOC*H*=CHS *cis*), 5.66 (d, 1H, $^3J_{HH}$ = 12 Hz, OCOCH=C*H*S *cis*), 5.56 (d, 1H, $^3J_{HH}$ = 15 Hz OCOCH=C*H*S *trans*), 4.00-4.01 (m, 2H, OCH_2CH_2OCO), 3.29-3.62 (m, 50H, OCH_2CH_2O), 3.12 (s, 3H, CH_3O) 2.43-2.82 (m, 4H, $COCH_2CH_2SH$ pm. FT-IR max/cm^{-1} 2867 (CH), 1734 (C=O), 1700 (C=O), 1100 (CO), 802 (C=C *cis*). ^{1}H NMR spectroscopy indicated *ca.* 91% *cis* isomer selectivity.

Model PEG thiol-yne reaction in H_2O (100%): ^{1}H NMR ($CDCl_3$, 300 MHz): δ 7.73 (d, 1H, $^3J_{HH}$ = 15 Hz, OCOC*H*=CHS *trans*), 7.43 (d, 1H, $^3J_{HH}$ = 12 Hz, OCOC*H*=CHS *cis*), 5.86 (m, 2H, OCOCH=C*H*S), 4.19-4.21 (m, 2H, OCH_2CH_2OCO), 3.44-3.79 (m, 50H, OCH_2CH_2O), 3.28 (s, 3H, CH_3O) 2.64-2.77 (m, 4H, $COCH_2CH_2SH$) ppm. FT-IR max/cm^{-1} 2868 (CH), 1735 (C=O), 1700 (C=O), 1099 (CO), 805 (C=C *cis*). ^{1}H NMR spectroscopy indicated *ca.* 93% *cis* isomer selectivity.

As stated in the main text, from herein onwards, % *cis* content of the gels are referred to from the values determined by FT-IR spectroscopy.

1.9 Gel fraction (GF)

To determine the gel fraction (GF) of the organogels, samples were lyophilized and their weights (W_g) recorded. The gels were then allowed to swell in deionized water for 3 d, with frequent water changes to extract any unreacted PEG precursors. After that period, the gels were lyophilized, and their weights (W_r) were recorded again. All measurements were repeated in triplicate. GF is expressed as:

$$\text{Gel Fraction (\%)} = \frac{\mathrm{W_r}}{\mathrm{W_g}} \times 100\ \% \qquad (1)$$

1.10 Equilibrium water content (EWC)

To determine the equilibrium water content (EWC), after the solvent exchange process, hydrogels were allowed to swell in PBS (pH 7.4) for 24 h, and their weight was recorded (W_s). Hydrogels were then lyophilized, and the weights recorded again (W_d). All measurements were repeated in triplicate. EWC is expressed as:

$$\text{EWC (\%)} = \frac{\mathrm{W_s} - \mathrm{W_d}}{\mathrm{W_s}} \times 100\% \qquad (2)$$

1.11 Swelling and degradation studies

Hydrogels (n = 3) were prepared as described above (section 1.5) and then placed in the corresponding solution (*i.e.* PBS or cell culture medium - MEM-α) at 37 °C in an orbital shaker-incubator (Model ES-20, Grant Instruments (Cambridge) Ltd.) with a shaking speed of 60 rpm. The swelling solution was replaced regularly to remove any degradation products and to prevent the build-up of solute concentration. At specific time points, the hydrogels were removed, gently blotted dry, and their weight was recorded. Hence, the swelling was monitored by the percentage of weight of the hydrogel at each time point (W_t) compared to the weight before submersion (W_0), which is defined as:

$$Swelling\ Factor\ (\%) = \frac{W_t}{W_0} \times 100\% \qquad (3)$$

1.12 Mesh size determination

Flory–Rehner calculations were used to determine each thiol–yne PEG hydrogel mesh size.[2] All measurements were run in triplicate, and the procedure followed was reported in detail in a previous study.[1a]

Fluorescence Recovery after Photobleaching (FRAP) is a very simple technique used to study dynamics, like diffusion rates, at the microscopic level. Here, we exploit this technique to assess the mass transport characteristic of the prepared hydrogels.[3] To that end, FTIC-dextran molecules, with molecular weights of 5 and 10 kDa, were used as macromolecular model compounds. Their hydrodynamic radii, r_H, which were calculated according to previous literature,[3a, 4] was determined to be of 1.35 and 1.90 nm, respectively. Hydrogels with a % *cis* content of 10%, 51%, and 100% were selected for this test (n = 3 for each % *cis* content) and immersed in aqueous solutions at 0.3 mg mL^{-1} of the respective FITC-dextran for 36 h, which allowed for FTIC-dextran molecules to diffuse into the hydrogel network.

The FRAP experiments, which were performed on an Olympus Confocal Laser Scanning Microscope (Fluoview FV3000; software FV31S-SW version 2.3.1.163), were based on the procedure described by Brandl *et al.*[3a] Briefly, all bleaching experiments were performed using

the 488 nm-line. After the area of interest was brought into focus (4.0X objective Lens Mag.), a time-series of digital images with a resolution of 1024 × 1024 pixel was recorded. After the acquisition of six pre-bleach images, a uniform disk with a diameter of 32.24 μm was bleached at maximum laser intensity (100% transmission). The bleaching phase happened quickly enough to avoid fluorescence recovery during bleaching. Immediately after bleaching, a stack of at least 30 images (between 30 and 80) was acquired in order to measure the recovery of fluorescence inside the bleached area. Several areas were analysed throughout the hydrogels. The experimental recovery curves were extracted from the images by the software (OLYMPUS cellSens Dimension Desktop 1.18). Indeed, curves that plotted the mean fluorescence intensities inside the bleached region, $I_{frap}(t)$, and inside a reference region, $I_{ref}(t)$, *versus* time were obtained from the stacked images (at least 12 curves for each condition). Next, $I_{frap}(t)$ was normalized to the prebleach intensity, $I_{frap}(\text{pre})$, and corrected for any bleaching effects that might have occurred during image acquisition:

$$f\,(t) = \frac{I_{\text{ref}}(\text{pre})}{I_{\text{ref}}\,(t)}\,\frac{I_{\text{frap}}(t)}{I_{\text{frap}}(\text{pre})} \qquad (4)$$

$f(t)$ is the normalized fluorescence intensity inside the bleached region, and $I_{ref}(\text{pre})$ is the fluorescence intensity inside the reference region before bleaching. In the following step, $f(t)$ was further normalized to the full scale using:

$$F(t) = \frac{f(t) - f(0)}{f(pre) - f(0)} \qquad (5)$$

where $f(0)$ is the normalized fluorescence intensity immediately after bleaching, and $f(\text{pre})$ the normalized fluorescence intensity before bleaching. Finally, the characteristic diffusion time τ_D and the mobile fraction k were determined by a least-squares fit of the following expression to the experimental recovery curve:

$$F(t) = ke^{\frac{\tau_D}{2t}}\left[I_0\left(\frac{\tau_D}{2t}\right) + I_1\left(\frac{\tau_D}{2t}\right)\right] \qquad (6)$$

where I_0 and I_1 are the modified Bessel functions of the first kind of zero and first order.[4-5]The diffusion coefficient D was then calculated by:

$$D = \frac{w^2}{\tau_D} \qquad (7)$$

$D = w^2/\tau_D$, where w is the radius of the bleached spot. The fitting was performed using RStudio (version 1.1.453).

1.13 Differential scanning calorimetry (DSC)

The freshly prepared gels were placed *in vacuo* (*ca.* 100 mTorr) for 24 h to remove solvent and then sliced into thin discs (*ca.* 10 mg). The thermal characteristics of the dried gels were determined using differential scanning calorimetry (DSC) (STARe system DSC3, Mettler Toledo) from −100 – 100 °C at a heating rate of 10 K min^{-1} for two heating/cooling cycles unless otherwise specified. The glass transition temperature (T_g), cold crystallization temperature (T_{cc}), melting temperature (T_m), and enthalpy of melting (ΔH_m) were determined from analysis of the second heating cycle to ensure adequate removal of solvent.

1.14 Mechanical characterization: uniaxial compressive tests

All uniaxial compressive testing was performed on a M100-1CT Testometric fitted with a load cell of 1 kN. Hydrogel samples, which were prepared as described above (n = 8-10), were tested after the solvent exchange process. A preload force of 0.1 N was set, and each test was carried out at a compression velocity of 5 mm min^{-1}. Each gel was subjected to 98% strain to determine the ultimate compressive stress and strain. Data was analysed using winTest mechanical analysis software.

1.15 Rheological characterization

All rheology was performed on an Anton Parr MCR 302 rheometer fitted with a parallel plate configuration (diameter of 8 mm) and a sandblasted bottom plate to reduce the slippage of the sample. Amplitude sweeps were conducted applying a constant frequency of 10 rad s^{-1}, while the strain was ramped logarithmically from 0.01% to 10% keeping the normal force constant at 0.04 N. All measurements were repeated in triplicate, and representative charts are shown.

1.16 Cryogenic Scanning Electron Microscopy

Cryogenic scanning electron microscopy (cryo-SEM) was performed on ZEISS SUPRA 55-VP equipped with cold stage and sample preparation chamber. Hydrogel samples were carefully placed on a stub and frozen in liquid nitrogen (-195 °C) under vacuum. The stub was then transferred to the cold stage (-125 °C) of the preparation chamber. There, the frozen sample was carefully surface fractured and sublimated at -95 °C for 15 min to reveal the cross-sectional surface. The temperature was then brought down to -125 °C, and the sample was sputter coated with platinum before being transferred under vacuum into the main SEM chamber (kept at -186 °C for imaging). The accelerating voltage was set at 2 kV to avoid burning the sample.

1.17 Biocompatibility studies: cytotoxicity of $4_{2A}4_{2S}$ hydrogels and degradation products

To confirm that our click-hydrogels and their degradation products were non-cytotoxic, cell viability tests on MC3T3 (murine pre-osteoblasts) and Y201 hTERT-immortalised human clonal mesenchymal stem cell (MSCs) were undertaken. MC3T3 cells were obtained from Public Health England and were cultured in 175 cm^2 tissue culture flasks using MEM alpha medium (Gibco), as advised by the supplier, with addition of 10% FBS and 1% pen/strep, at 37 °C, 5% CO_2. Similarly, Y201 MSCs[6], which were a kind gift from Prof Paul Genever (University of York) were cultured in MEM-α medium supplemented with 10% v/v FCS, 1% penicillin/streptomycin, 10 µM asc-2-phos, and 5 mL Glutamax.

Hydrogels (n = 6-12) were prepared as described above and then placed in a fibronectin solution (25 µg ml^{-1} in PBS) overnight (Fibronectin Bovine Protein, Plasma – Cat. Number 33010018 – Invitrogen by Thermo Fischer Scientific – Lot 198036). Specifically, hydrogels with a % *cis* content of 10%, 51%, and 100% were selected for this test, while hydrogels made using PBS as solvent were also included as control. After such amount of time, hydrogels were carefully rinsed with PBS. Cells were seeded on a 2D configuration on the top surface of the click-hydrogels (127k cells/cm^2) and left to adhere for 3 h before adding 1 mL of cell culture media. Cells proliferated for 7 d, and culture media was replaced with fresh one every two d during the whole incubation period. Cell viability was measured at specific time points using PrestoBlue® Cell Viability Reagent (Invitrogen™) or Alamar Blue® (Invitrogen™) for MC3T3 and Y201 cells, respectively, following the supplier's protocol. Cell viability was also assessed using Live/Dead™ Viability/Cytotoxicity Kit (Invitrogen™), which includes calcein AM for live cells (λEx. = 495, λEm. = 515) and ethidium homodimer for dead cells (λEx. = 528, λEm. = 617). The

staining solution was prepared by dissolving calcein AM (0.5 µL mL^{-1}) and ethidium homodimer (2 µL mL^{-1}) in PBS and incubated with the samples for 30 min. After washing in PBS, samples were imaged using an Olympus Confocal Laser Scanning Microscope (Fluoview FV3000) and excited using the 488 and 561 nm lasers. Images were processed using CellSens (Olympus) and ImageJ software (1.52i).

For cell growth and morphology monitoring, after specific time points, cells were washed with PBS (1x, 5 min) and fixed using 4% paraformaldehyde (20 min, 22 °C). After washing with PBS (3x, 5 min each), permeabilization was carried out using 0.5% Triton X-100 (15 min, 22 °C) and samples were washed again with PBS (3x, 5 min each). Alexa Fluor™ 488 Phalloidin (Thermo Fisher Scientific, A12379) was used to stain filamentous actin (1:40 dilution, 1 h, 22 °C) and, subsequently, DAPI was used to stain cell nuclei (1:1000, 10 min, 22 °C). Cells were imaged using DAPI (wavelength = 405/443-483 nm excitation/emission) and Alexa Fluor™ 488 (wavelength = 488/500-540 nm excitation/emission) filters. Images were processed using CellSens (Olympus) and ImageJ software (1.52i).

1.18 Reporting mechanoresponsive behaviour

The Y201 MSC line was cultured in MEM-α medium supplemented with 10% v/v FCS, 1% penicillin/streptomycin, 10 µM ascorbate-2-phosphate, and 5 mL Glutamax. For assessing their mechanoresponsive behaviour, hydrogels were prepared as described above (section 1.15), and Y201 MSCs were seeded on top at 2.4k/cm^2. After 72 h of incubation, cells were fixed, permeabilized and stained with DAPI and Alexa Fluor™ 488 Phalloidin (as above). Cells were imaged using DAPI (wavelength = 405/443-483 nm excitation/emission) and Alexa Fluor™ 488 (wavelength = 488/500-540 nm excitation/emission) filters. Images were processed using CellSens (Olympus) and ImageJ software (1.52i). Morphometric data was extracted from the images after analysing at least 140 cells per condition using ImageJ software (1.52i).

Similarly, after 7 d incubation, cells were fixed, permeabilized and stained with DAPI and Alexa Fluor™ 488 Phalloidin (as above), as well as YAP1 primary antibody (Rabbit Polyclonal; Cat. number: 13584-1-AP; Proteintech®; 300 µg/mL)/Alexa Fluor™ 594 donkey anti-rabbit secondary antibody (Cat. Number R37119; readyProbes™ reagent - Invitrogen by Thermo Fischer Scientific). In this case, the procedure followed was: cells were washed with PBS (1x, 5 min) and fixed using 4% paraformaldehyde (20 min, RT). After washing with PBS (3x, 5 min each), permeabilization was carried out using 0.5% Triton X-100 (15 min, 22 °C) and samples were washed again with PBS (3x, 5 min each). Blocking was performed by immersing the samples in 2% BSA (0.25% Triton X-100 in PBS) for 2 h at RT. After washing with PBS (1x, 5 min), the surface of the hydrogels was covered with 40 µL of YAP1 Ab solution (dilution factor of 1:50 in 1% BSA (0.25% Triton X-100 in PBS)) and left overnight at 4 °C. The next morning, after washing with PBS (3x, 5 min each), the surface of the hydrogels was covered with 40 µL of Alexa Fluor™ 594 donkey anti-rabbit (1 drop in 0.5 mL of 1% BSA (0.25% Triton X-100 in PBS) for 1 at RT. Finally, after washing with PBS (3x, 5 min each), Alexa Fluor™ 488 Phalloidin was used to stain filamentous actin (1:40 dilution, 1 h, 22 °C) and, subsequently, after washing (3x, 5 min each), DAPI was used to stain cell nuclei (2 µL in 1000, 10 min, 22 °C). Cells were imaged using DAPI (wavelength = 405/443-483 nm excitation/emission), Alexa Fluor™ 488 (wavelength = 488/500-540 nm excitation/emission) and Alexa Fluor™ 594 (wavelength = 561/650-680 nm excitation/emission) filters. Images were processed using CellSens (OLYMPUS cellSens Dimension Desktop 1.18) and ImageJ software (1.52i).

1.19 Statistical Analysis

One-way analysis of data was used for analyzing the data when appropriate. The data represents the mean and standard deviation. Quantified data were categorized as significantly different when $p < 0.05$ using Tukey's[7] multiple comparison test.

SUPPLEMENTARY DATA

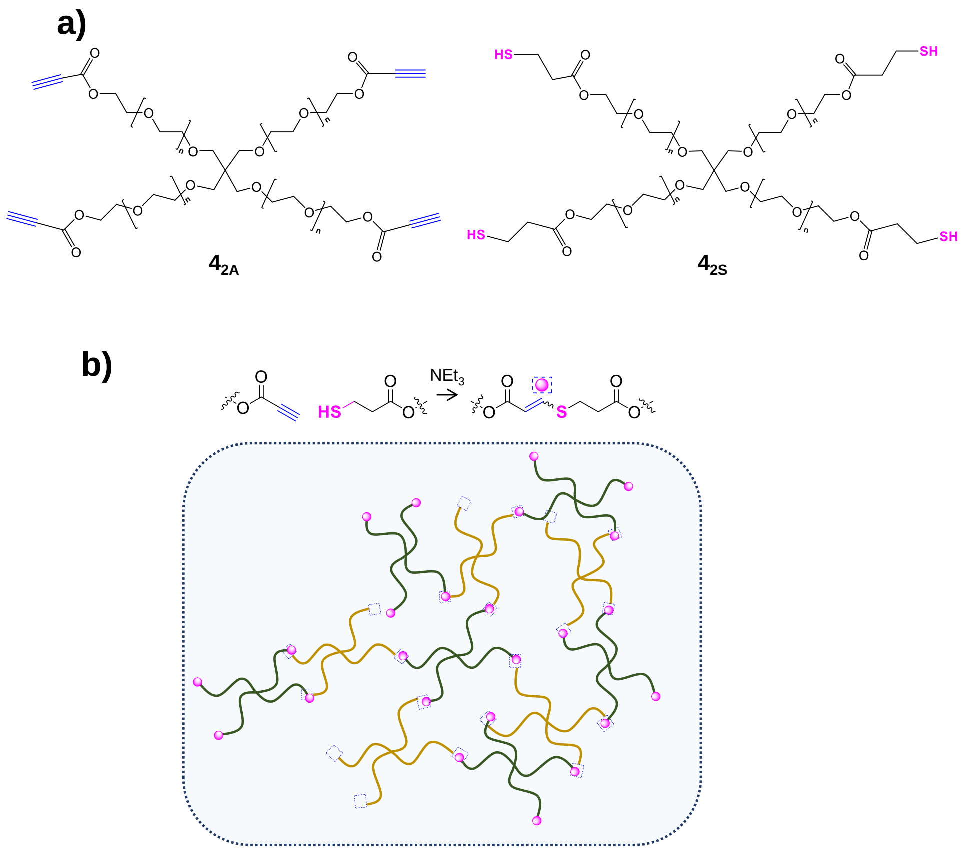

Figure S1. a) PEG precursors synthesized to prepare thiol-yne click-hydrolgels. b) Generic nucleophilic thiol-yne reaction between 4_{2A} and 4_{2S} and schematic of the resulting hydrogel network.

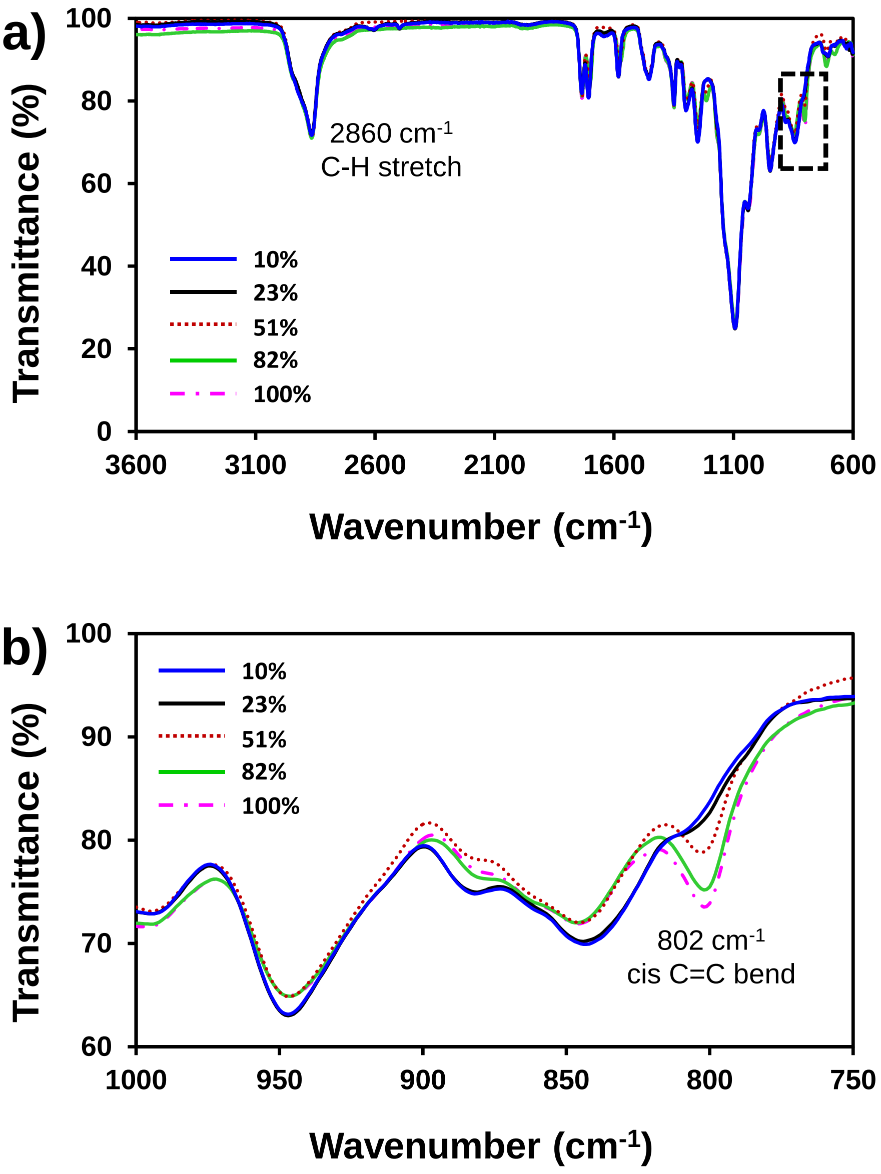

Figure S2. FT-IR spectra obtained for stereochemically controlled PEG-based click-hydrogels.

We determined the *cis*:*trans* ratio of the obtained click-networks by means of FT-IR spectroscopy (Figure S2). For each organogel, at least 10 measurements were performed on different areas of the disk. Additionally, FT-IR spectroscopy gave an insight into the reaction conversion: the alkyne stretching signal (*ca.* 2100 cm^{-1}) disappeared after reacting with the thiol-moiety. The % *cis* content in the organogel was determined by normalising the area of the bending signal ascribed to the cis alkene (802 cm^{-1}) with the area of the stretching signal assigned to C-H bond (2866 cm^{-1}). When using H_2O as the gelation solvent, the resulting *cis* content was 94%. Therefore, relative to this value, the *cis* content for the organogels was determined by dividing the normalised peak area by the area of the material synthesised using aqueous conditions. As a result, the careful optimization of the solvent conditions during gelation yielded a series of thiol-yne PEG organogels with a range of different *cis*:*trans* ratio values (*i.e.* 10, 23, 51 and 82% *cis* content). Interestingly, even in highly non-polar conditions (*i.e.* $CHCl_3$), a *cis* content of 10% was still present.

Additionally, a model compound study was used to elucídate the relative stereochemistry of the $\mathbf{4_{2A}4_{2S}}$ network by ^{1}H NMR spectroscopy. To that end, a methyl ether PEG (MeO-PEG, 550 g mol^{-1}) was monofunctionalised with an alkyne or a thiol end group by Fisher esterification, which resulted in cross-linking precursors with high conversion (> 92%) as determined by ^{1}H NMR spectroscopy and SEC analysis (Figures S3 and S4). The alkyne- and thiol-functionalised MeO-PEG precursors were dissolved in the appropriate solvent system, and triethylamine was added. The thiol-yne reaction was then sealed and stirred for 1 h before concentrating under vacuum. After the solvent was removed, the product was analysed through ^{1}H NMR spectroscopy. The signals corresponding to the *cis* and *trans* protons, which are distinguishable and well resolved in the ^{1}H NMR spectrum (Figure S5 and Figure S6), were assigned using the three bond coupling constants ($^{3}J_{HH}$). Indeed, these constants differ depending on the stereochemistry of the alkene bond: $^{3}J_{HH}$ values for the *trans* configuration range between 11-19 Hz, whereas $^{3}J_{HH}$ values for the *cis* range between 5-14 Hz. Hence, for the vinyl thioether compound, the two doublets at δ = 6.95 and 5.65 ppm were assigned to the *cis* isomer (*i.e.* $^{3}J_{HH}$ = 9 Hz), while the signals at δ = 7.42 and 5.55 ppm corresponded to the *trans* isomer since the coupling constant was greater (*i.e.* $^{3}J_{HH}$ = 15 Hz), and the relative *cis*:*trans* ratio was calculated accordingly. In good agreement with previous studies, the increase in polarity of the reaction solvent led to a higher % *cis* content.[8] Overall, even though the % *cis* content values determined by FT-IR and ^{1}H NMR spectroscopy exhibit some discrepancies, probably arising from experimental differences between both techniques, our approach produced $\mathbf{4_{2A}4_{2S}}$ click-hydrogels with controlled stereochemistry by fine adjustment of the gelation conditions only, thus maintaining the polymer concentration, as well as the cross-linking density, constant throughout the systems. From herein onwards, we will identify the % *cis* content of the hydrogels referring to the values determined by FT-IR spectroscopy.

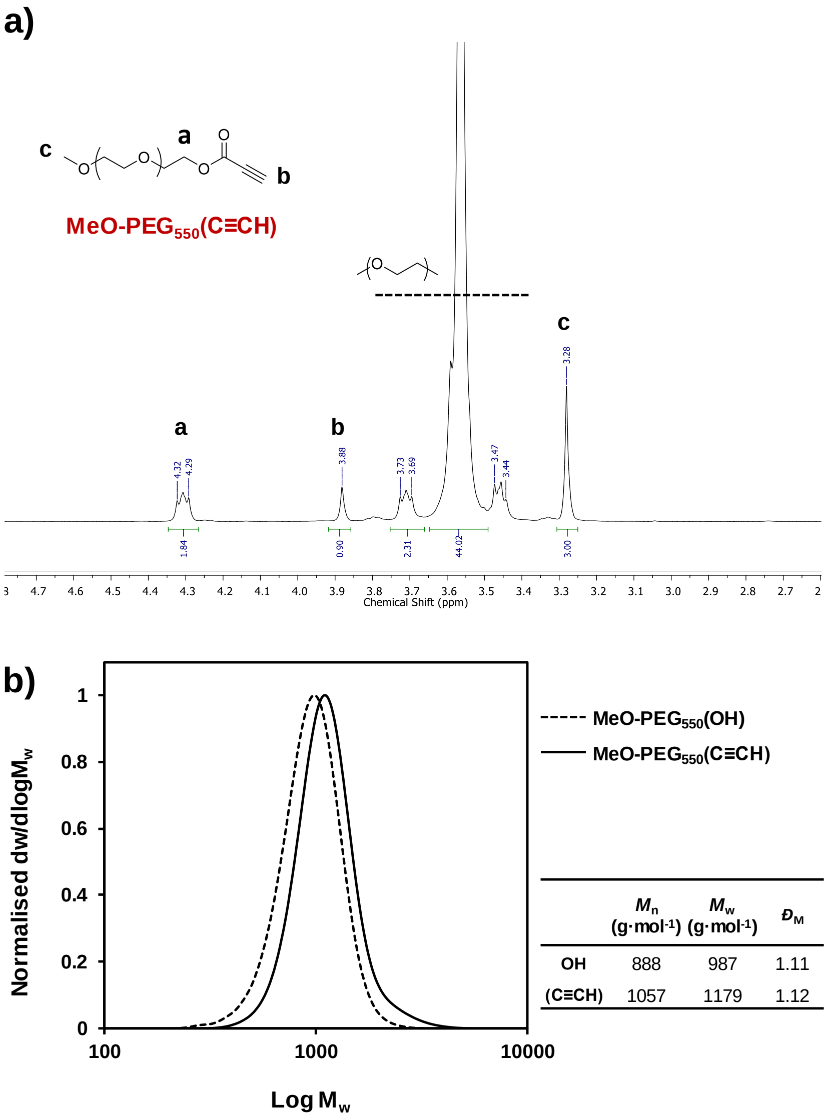

	M_n (g·mol⁻¹)	M_w (g·mol⁻¹)	$Đ_M$
OH	888	987	1.11
(C≡CH)	1057	1179	1.12

Figure S3. a) ^{1}H NMR spectrum of MeO-PEG$_{550}$(C≡CH) in d$_6$-acetone (300 MHz, 298 K). b) SEC chromatograms of MeO-PEG$_{550}$(OH) and MeO-PEG$_{550}$(C≡CH). Molecular weight determined against poly(methyl methacrylate) using DMF (5 mM NH_4BF_4) as eluent.

a)

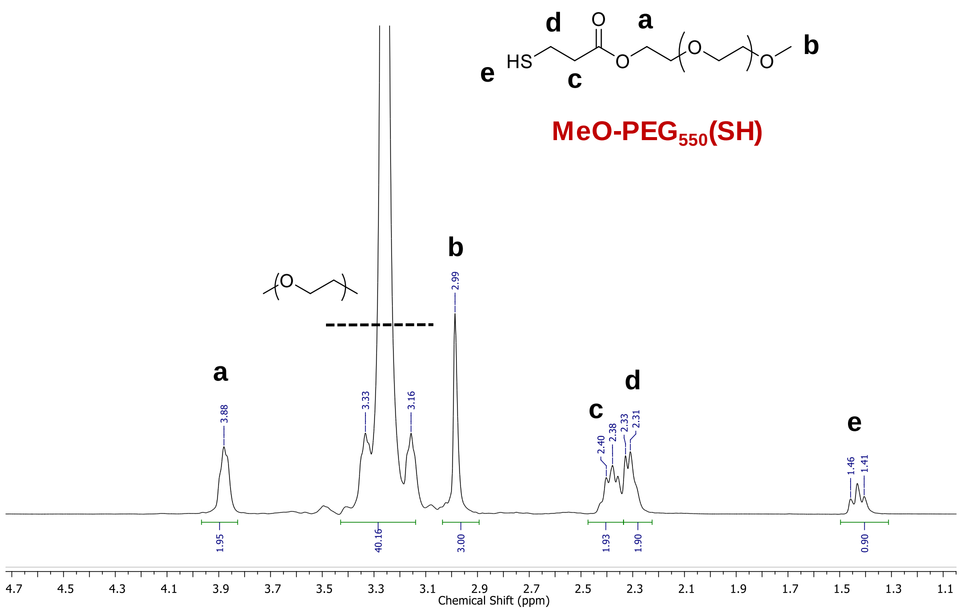

b)

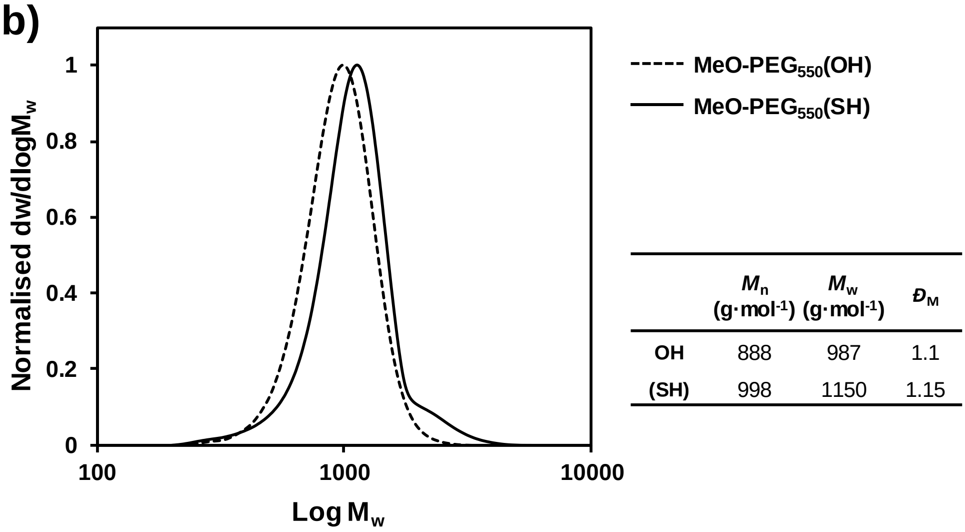

	M_n (g·mol[-1])	M_w (g·mol[-1])	$Đ_M$
OH	888	987	1.1
(SH)	998	1150	1.15

Figure S4. a) ^{1}H NMR spectrum of MeO-PEG$_{550}$(SH) in $CDCl_3$ (300 MHz, 298 K). b) SEC chromatograms of MeO-PEG$_{550}$(OH) and MeO-PEG$_{550}$(SH). Molecular weight determined against poly(methyl methacrylate) using DMF (5 mM NH_4BF_4) as eluent.

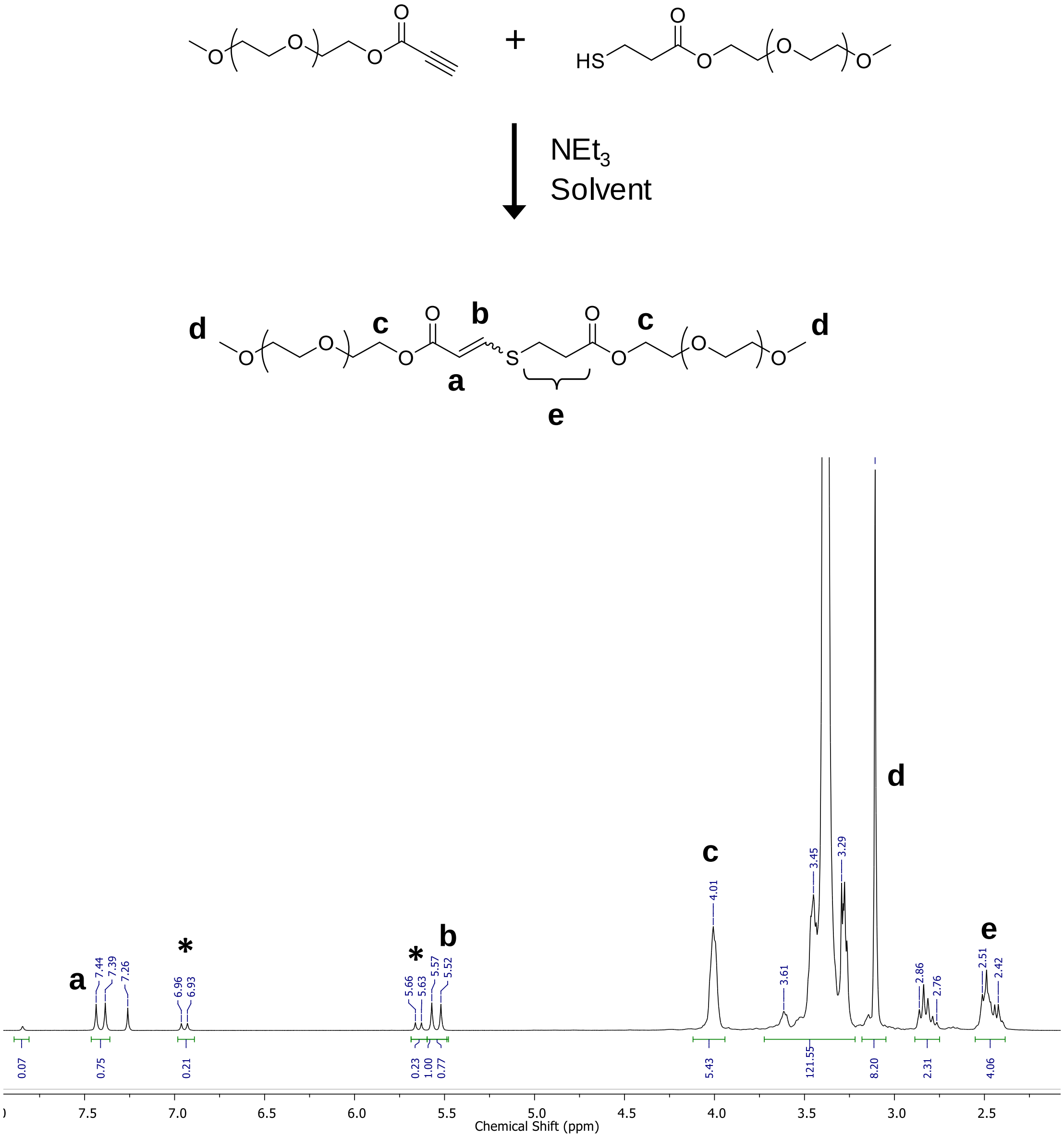

Figure S5. Nucleophilic thiol-yne reaction between monofunctionalised alkyne- and thiol-PEG precursors. The solution was stirred at room temperature (23 °C) for 1 h. After such amount of time, the solvent was removed *in vacuo* and the product was characterized by ^{1}H NMR spectroscopy (thiol-yne product obtained using $CHCl_3$ as solvent system; $CDCl_3$, 300 MHz, 298 K). * = *cis* configuration.

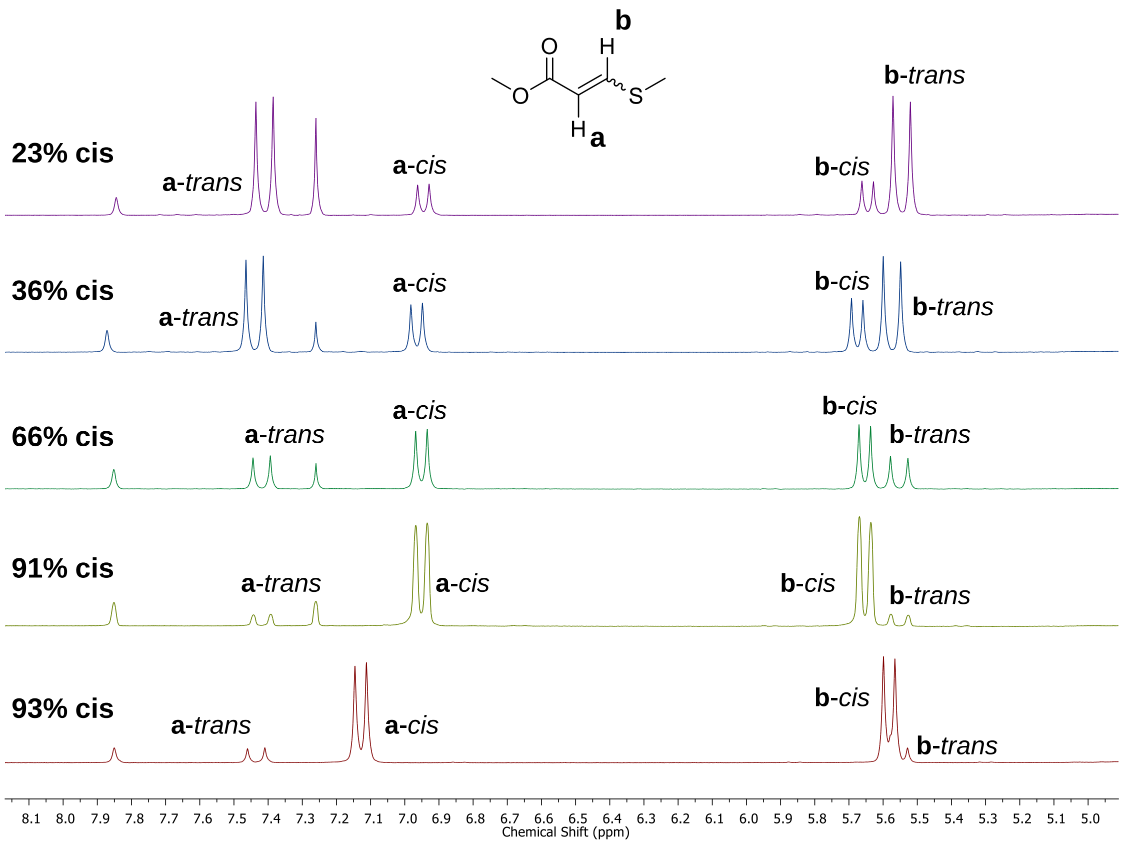

Figure S6. ^{1}H NMR spectra of the vinyl thioether product obtained following different reaction conditions as specified in Table S1 ($CDCl_3$, 300 MHz, 298 K; $(CD_3)_2CO$, 300 MHz, 298 K for H_2O system). % *cis* content values as determined by ^{1}H NMR spectroscopy.

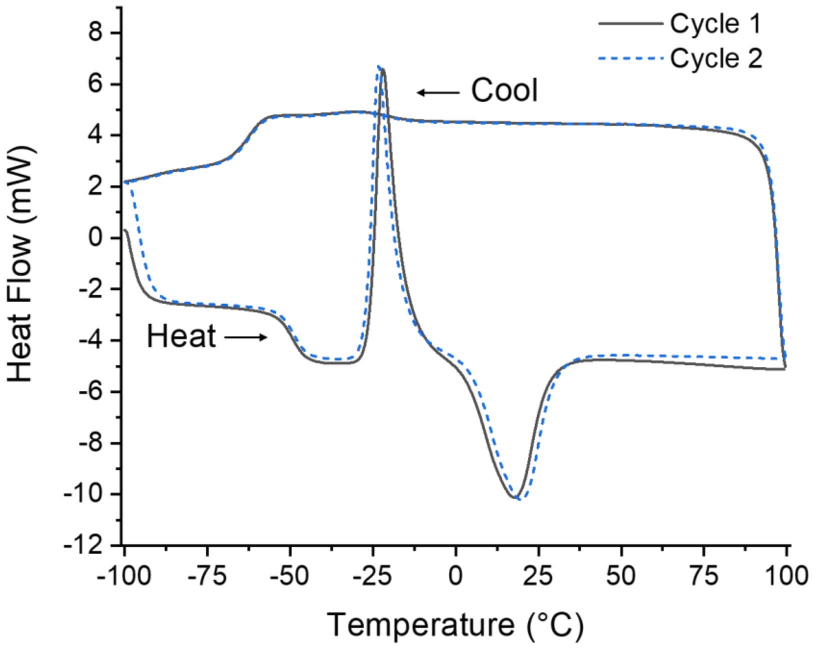

Figure S7. DSC thermograms of two heating/cooling cycles for dried 10% *cis* gel tested at 10 °C min^{-1} from −100 to 100 °C.

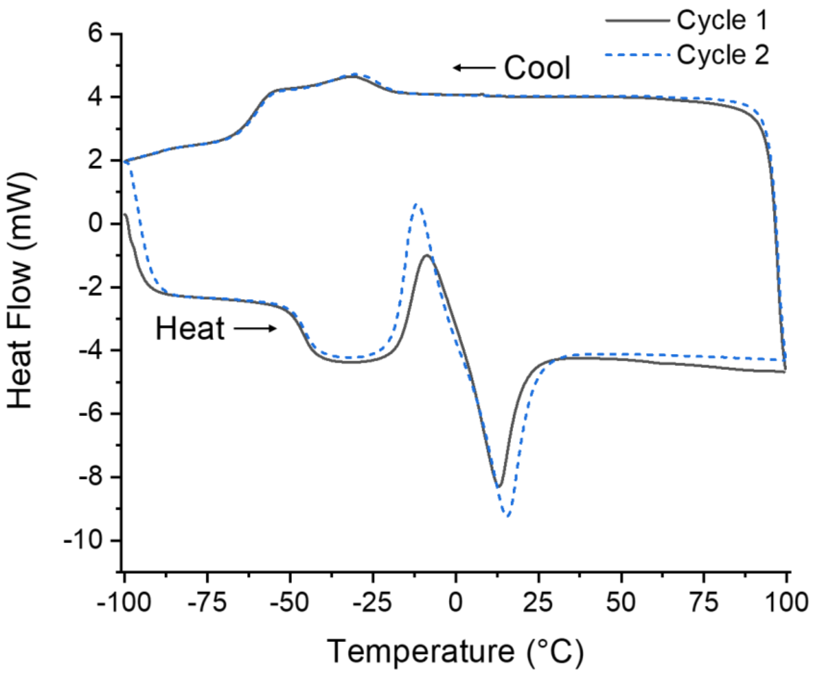

Figure S8. DSC thermograms of two heating/cooling cycles for dried 23% *cis* gel tested at 10 °C min^{-1} from −100 to 100 °C.

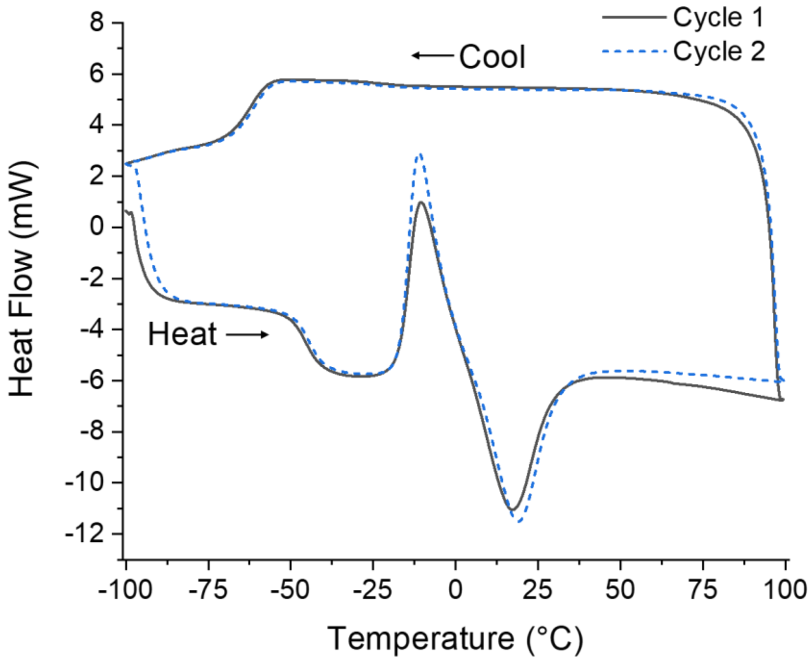

Figure S9. DSC thermograms of two heating/cooling cycles for dried 51% *cis* gel tested at 10 °C min^{-1} from −100 to 100 °C.

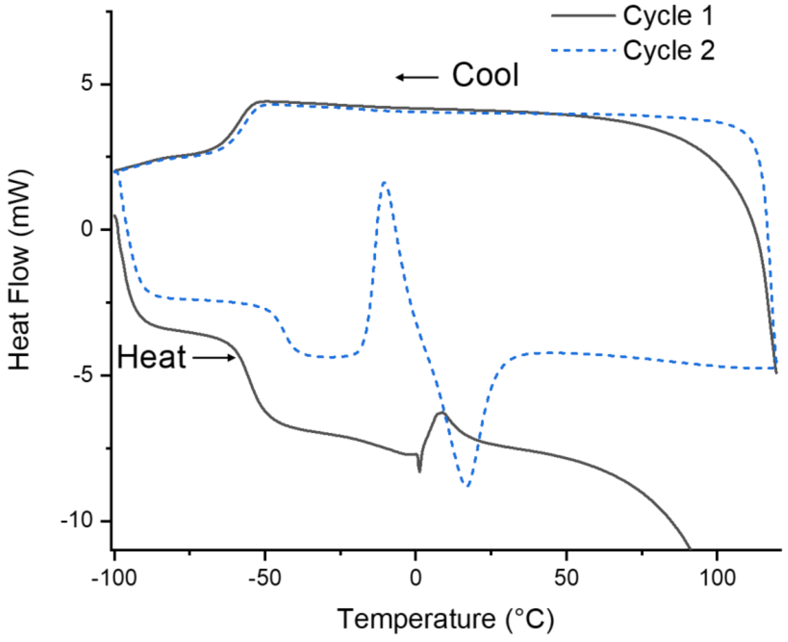

Figure S10. DSC thermograms of two heating/cooling cycles for dried 82% *cis* gel tested at 10 °C min^{-1} from −100 to 120 °C. After the 1st heating cycle, the sample was held at 120 °C for 5 minutes to remove residual solvent.

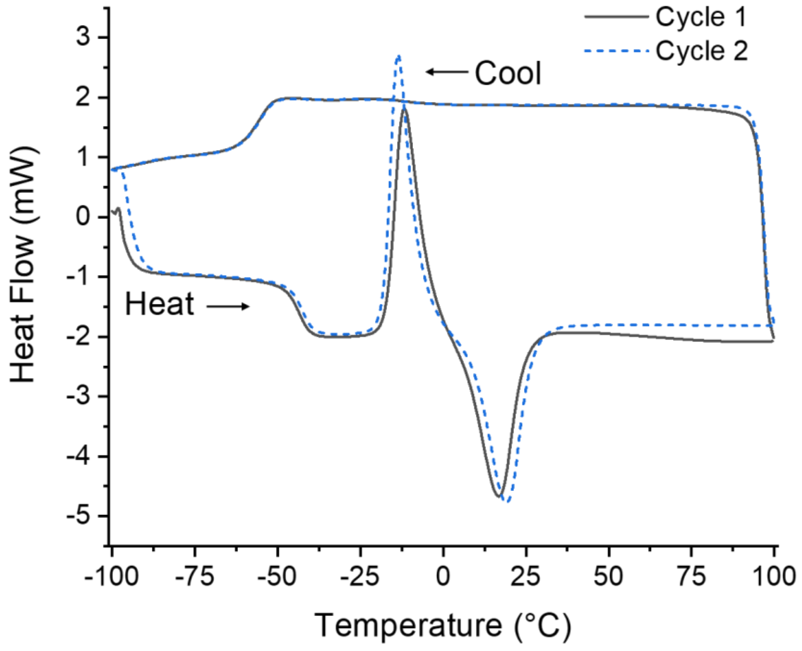

Figure S11. DSC thermograms of two heating/cooling cycles for dried 100% *cis* gel tested at 10 °C min^{-1} from −100 to 100 °C.

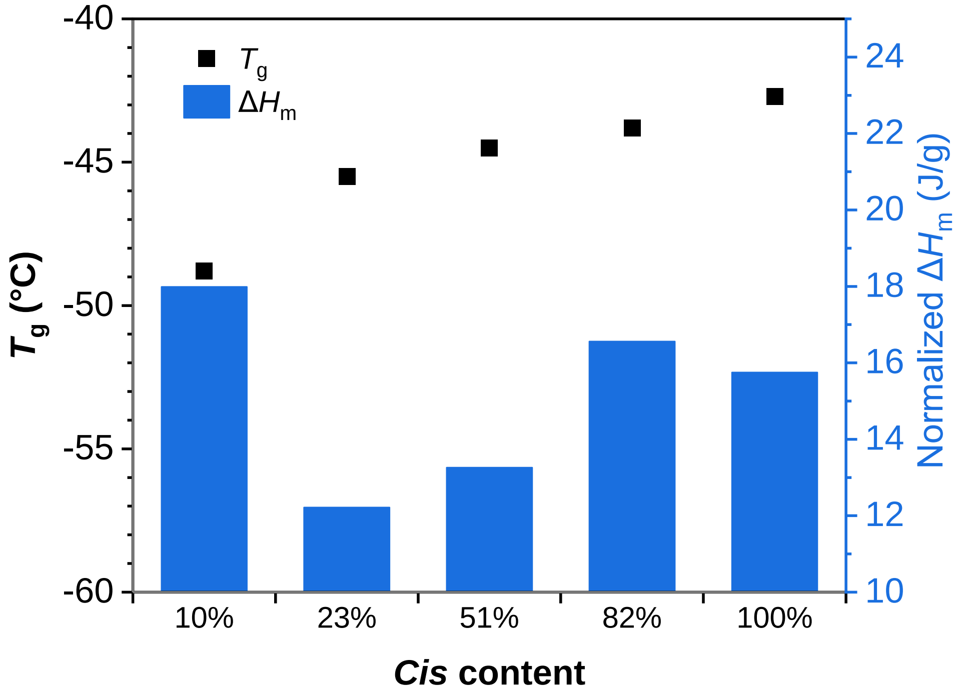

Figure S12. Bar plot of glass transition temperature (T_g) and normalized enthalpy of melting (ΔH_m) for dried gels calculated from 2nd heating cycle of DSC thermograms. Samples tested at 10 °C min^{-1}.

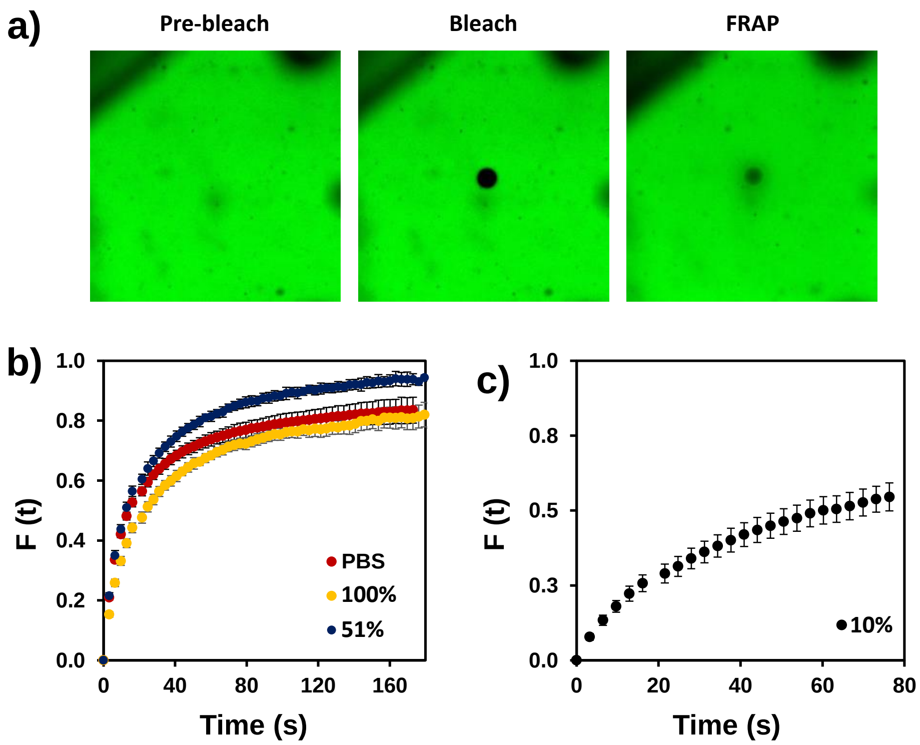

Figure S13. Mesh size determination by FRAP: a) Images of a typical FRAP experiment (100% *cis* hydrogels, FITC-dextran 10 kDa). Pre-bleach image shows the sample before bleaching (left image); a uniform disk is bleached during the Bleach step (middle image); finally, the laser intensity is reduced, and fluorescence recovery is recorded over time (FRAP step, right image). b-c) Representative recovery curves obtained after the mathematical processing for each % *cis* content using FITC-dextran with 10 kDa (b) and 5 kDa (c).

Table S2. FRAP data determined for FTIC-dextran incoporated into hydrogels. Mesh size is deduced from the mass transport data. To the experimental data shown in Figure S13b and c, a least-squares fit was applied to obtain the mobile fraction (k) and the diffusion coefficient (D). If FTIC-dextran displays a mobile fraction (k) > 55%, it is assumed that the mesh size is similar to the molecule diameter.

**% *cis* content**	**FTIC-dextran (diameter)**	***k* (-)**	***D* (μm²/s)**	**Mesh Size (nm)**
100		80.9% ± 4.2%	15.9 ± 5.2	*ca.* 4
51	10 kDa (3.8 nm)	91.5% ± 1.9%	18.7 ± 0.9	*ca.* 4
10		N/A	N/A	< 4
10	5 kDa (2.7 nm)	60.8% ± 4.5%	5.0 ± 1.4	*ca.* 2.5

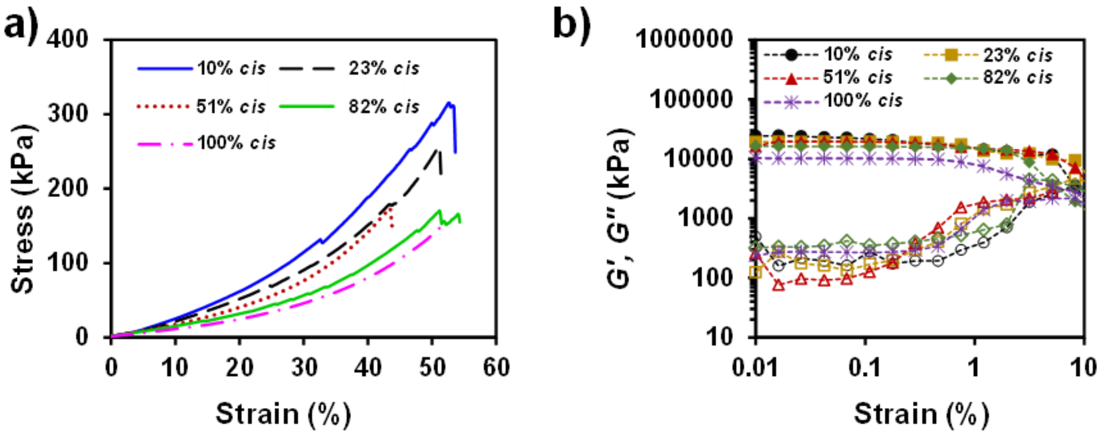

Figure S14. Mechanical characterization of stereochemically controlled hydrogels [Error bars: SD with n = 7-9]. a) Representative stress-strain curves for each % *cis* content. b) Representative amplitude sweep curves recorded from 0.1% to 10% at 10 rad·s^{-1}.

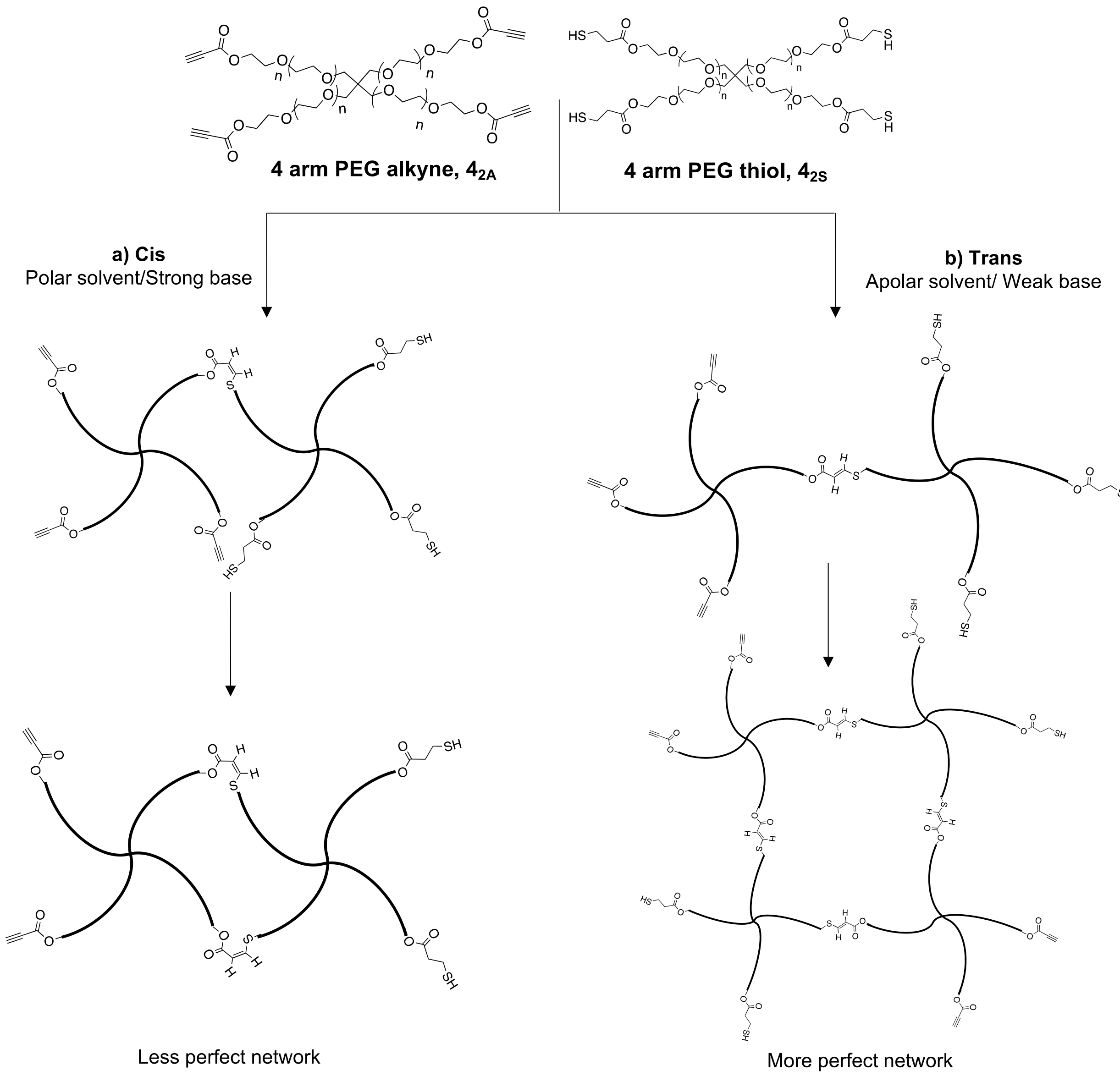

Figure S15: Schematic of network formation for *cis* versus *trans* bonds. a) *Cis* bond, functional groups brought into closer proximity, increasing the probability of loop defect formation (*i.e.* intramolecular reaction), decreasing network formation, resulting in softer click-hydrogels. b) *Trans* bond, functional groups further apart, increasing the probability that a bond will be formed with another PEG molecule (*i.e.* intermolecular reaction), increasing network formation, resulting in stiffer click-hydrogels.

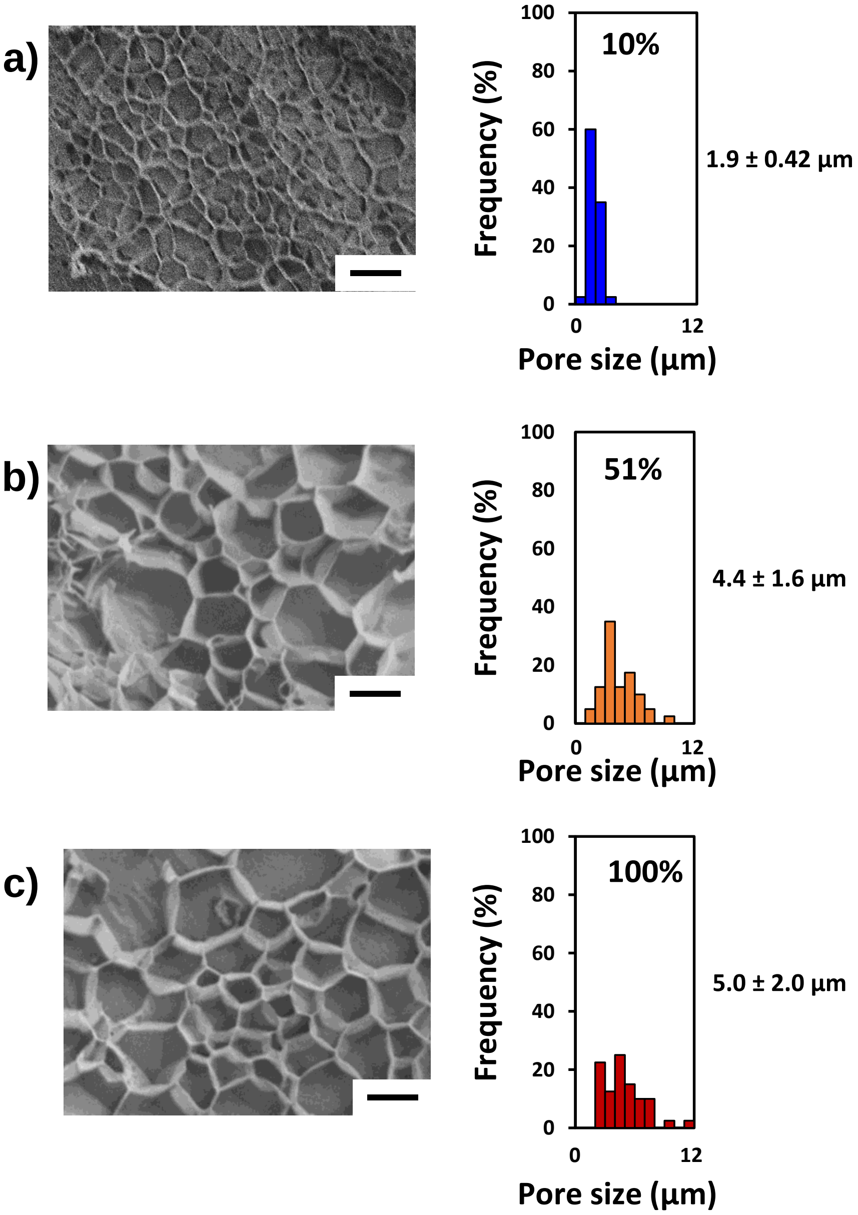

Figure S16. Right column: cyro-SEM images of click-hydrogels prepared with a) 10%, b) 51%, and c) 100% *cis* content (scale bar 4 µm). Left column: pore size distribution and average ± SD.

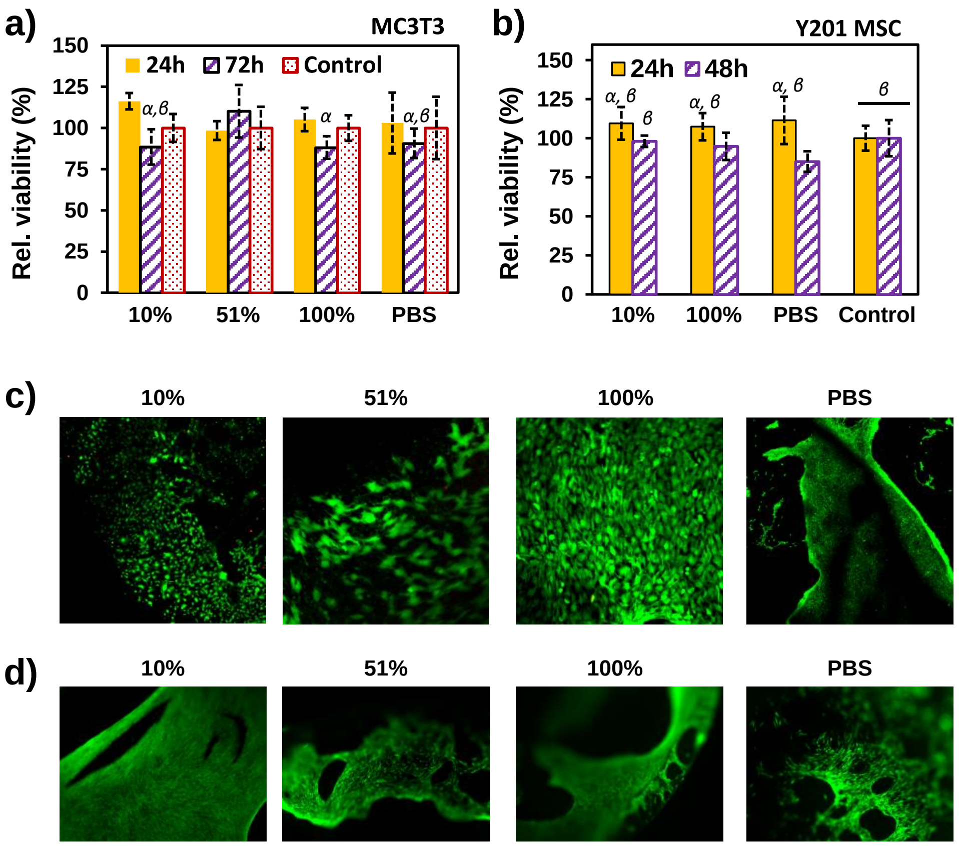

Figure S17. Cytocompatibility of of click-hydrogels: a) Cytotoxicity of the degradation products released from hydrogels prepared with 10%, 51%, and 100% *cis* content, as well as PBS, that had been immersed in cell culture media at 37 °C for 24 h and 72 h. MC3T3 cells were incubated with that media and fresh media (control), and viability (in % relative to control) was determined at time point 96 h. Greek letters on the bars refer to significant differences (p-value <0.05): *α vs* 10% *cis* content at 24 h; *β vs* 51% *cis* content at 72 h. b) Cytotoxicity of the degradation products released from hydrogels prepared with 10% and 100% *cis* content, as well as PBS, that had been immersed in cell culture media at 37 °C for 24 h and 48 h. Y201 MCS were incubated with that media and fresh media (control), and viability (in % relative to control) was determined at time point 96 h. Greek letters on the bars refer to significant differences (p-value <0.05): *α vs* 100% *cis* content at 48 h; *β vs* PBS at 48 h. c-d) Live/Dead images taken for MC3T3 (c) and Y201 MSCs (d) seeded on top of the hydrogels and incubated for 7 days.

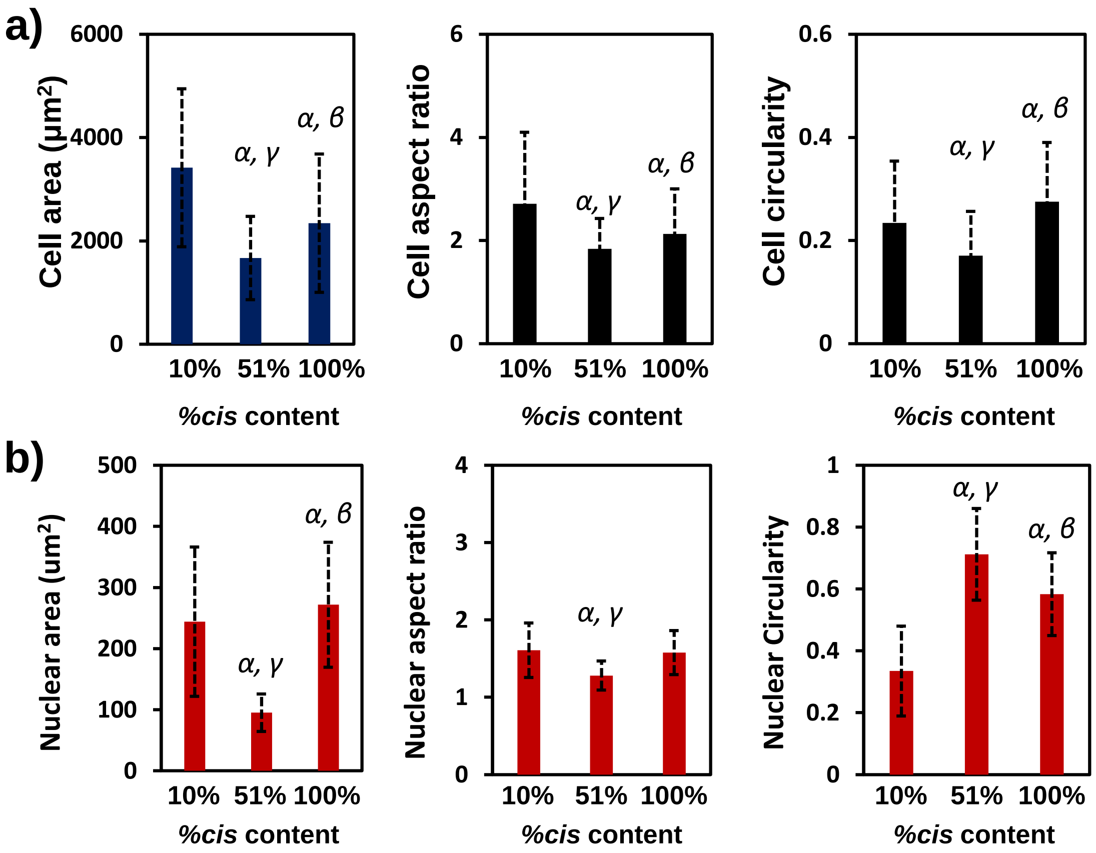

Figure S17. Morphometric data of Y201 MSCs seeded on stereocontrolled thiol-yne PEG hydrogels. Greek letters refer to significant differences (p-value <0.05): *α vs* 10%; *β vs* 51%; and *γ vs* 100% cis content.

AUTHOR CONTRIBUTIONS

L.J.M., M.M.P.M., S.M.R. and A.P.D. conceived and designed the project. L.J.M. and M.M.P.M. synthesised and characterised the materials. J.C.W. performed thermal analyses of the materials. C.S. measured and analysed FT-IR data. J.E.S. and M.M.P.M. conducted the cell experiments and maintained cell lines. The paper was written through contributions from L.J.M., M.M.P.M., J.C.W, S.M.R. and A.P.D. All authors have given approval to the final version of the paper.

REFERENCES

[1] aL. J. Macdougall, M. M. Perez-Madrigal, M. C. Arno, A. P. Dove, *Biomacromolecules* **2018**, *19*, 1378-1388; bL. J. Macdougall, M. M. Perez-Madrigal, J. E. Shaw, M. Inam, J. A. Hoyland, R. O'Reilly, S. M. Richardson, A. P. Dove, *Biomater. Sci.* **2018**, *6*, 2932-2937.

[2] aS. P. Zustiak, J. B. Leach, *Biomacromolecules* **2010**, *11*, 1348–1357; bT. Canal, N. A. Peppas, *J. Biomed. Mater. Res. A* **1989**, *23*, 1183-1193

[3] aF. Brandl, F. Kastner, R. M. Gschwind, T. Blunk, J. Teβmar, A. Göpferich, *J Controlled Rel.* **2010**, *142*, 221-228; bM. H. Hettiaratchi, A. Schudel, T. Rouse, A. J. Garcia, S. N. Thomas, R. E. Guldberg, T. C. McDevitt, *APL Bioeng.* **2018**, *2*, 026110; cE. Schuster, J.

Eckardt, A.-M. Hermansson, A. Larsson, N. Lorén, A. Altskär, A. Ström, *Soft Matter* **2014**, *10*, 357-366; dA. Carisey, M. Stroud, R. Tsang, C. Ballestrem, in *Cell migration: Developmental methods and protocols* (Eds.: C. M. Wells, M. Parsons), Humana Press, Totowa, NJ, **2011**, pp. 387-402.

[4] K. Braeckmans., L. Peeters, N. N. Sanders, S. C. D. Smedt, J. Demeester, *Biophys. J.* **2003**, *85*, 2240–2252.

[5] D. M. Soumpassis, *Biophys. J.* **1983**, *41*, 95-97.

[6] S. James, J. Fox, F. Afsari, J. Lee, S. Clough, C. Knight, J. Ashmore, P. Ashton, O. Preham, M. Hoogduijn, Raquel De Almeida R. Ponzoni, Y. Hancock, M. Coles, P. Genever, *Stem Cell Rep.* **2015**, *4*, 1004-1015.

[7] J. W. Tukey, *Biometrics* **1949**, *5*, 99-114.

[8] V. X. Truong, A. P. Dove, *Angew. Chem. Int. Ed.* **2013**, *52*, 4132-4136.
